# Endovascular Electrodes for Electrical Stimulation of Blood Vessels for Vasoconstriction – a Finite Element Simulation Study

**DOI:** 10.1038/srep31507

**Published:** 2016-08-18

**Authors:** Noa Kezurer, Nairouz Farah, Yossi Mandel

**Affiliations:** 1 Mina and Everard Goodman Faculty of Life Sciences, Optometry and Visual Science Track and Bar-Ilan’s Institute for Nanotechnology and Advanced Materials (BINA), Bar-Ilan University, Ramat-Gan, Israel

## Abstract

Hemorrhagic shock accounts for 30–40 percent of trauma mortality, as bleeding may sometimes be hard to control. Application of short electrical pulses on blood vessels was recently shown to elicit robust vasoconstriction and reduction of blood loss following vascular injury. In this study we present a novel approach for vasoconstriction based on endovascular application of electrical pulses for situations where access to the vessel is limited. In addition to ease of access, we hypothesize that this novel approach will result in a localized and efficient vasoconstriction. Using computer modeling (COMSOL Multiphysics, Electric Currents Module), we studied the effect of endovascular pulsed electrical treatment on abdominal aorta of pigs, and compared the efficiency of different electrodes configurations on the electric field amplitude, homogeneity and locality when applied on a blood vessel wall. Results reveal that the optimal configuration is the endovascular approach where four electrodes are used, spaced 13 mm apart. Furthermore, computer based temperature investigations (bio-heat model, COMSOL Multiphysics) show that the maximum expected temperature rise is of 1.2 degrees; highlighting the safety of the four endovascular electrodes configuration. These results can aid in planning the application of endovascular pulsed electrical treatment as an efficient and safe vasoconstriction approach.

Hemorrhagic shock is a leading cause of trauma mortality accounting for 30–40 percent of cases due to the challenges of controlling bleeding in hard to reach vessels^1^,^2^. Uncontrolled bleeding also accounts for over 80 percent of deaths^2^ in the battlefield and is the most common preventable cause of death in the combat arena. The use of tourniquets has dramatically decreased the mortality rate in cases of major limb trauma^3^. However, uncontrolled bleeding caused by solid organ trauma or junctional bleeding from vessels where tourniquets cannot be used (such as cases of trauma to femoral artery) is still one of the main causes of death in the field. To date, there is no optimal method for controlling non-compressible hemorrhage onsite before reaching the hospital^3^,^4^,^5^. Moreover, even in a hospital setting, it remains challenging to control the bleeding in both elective (e.g. bypass procedure and femorofemoral shunt) and emergency (e.g. vessel repair following trauma) scenarios, as the surgeon has to extensively expose the vessel and mechanically clamp both the proximal and distal sides of the artery before beginning the operation. The need for vascular surgical intervention has increased dramatically in the last few decades^6^ and novel technologies, such as closed endovascular^6^,^7^ or laparoscopic^8^ approaches have been recently developed. This rising need and the aforementioned limitations of available techniques in non-compressible hemorrhage control created great interest in the developing of new techniques for eliciting vasoconstriction.

One such technique can rely on applying short electrical pulses for inducing vasoconstriction. The vasoconstrictive effect of electric current on blood vessels was previously observed^9^ and was further characterized in the thorough work of Palanker *et al*. in a model of chorioallantoic membranes of chicken embryos^10^. In addition, we have recently shown that short (μs-ms) electrical pulses delivered by extravascular electrodes elicited robust and controlled vasoconstriction in femoral and mesenteric artery of rats^11^,^12^. Vessel constriction was dependent upon pulse amplitude, duration and pulse repetition rate. Investigation of the recovery dynamics revealed that for certain pulse parameters the vasoconstriction is completely reversible while for other parameters (higher repetition rates and longer pulse durations) there is permanent occlusion and clot formation. Furthermore, we demonstrated that short electrical pulses reduced blood loss by 2.5^13^ and 7 fold from the liver and femoral artery bleeding^11^, respectively.

Notwithstanding, in all these studies electrical stimulation was delivered via extravascular electrodes under controlled experimental conditions where blood vessels’ exposure can be easily obtained. In some trauma or elective vascular surgery it is technically challenging to expose the vessel for the introduction of the electrodes. This technical challenge can be overcome by using endovascular electrodes, which can be introduced into the blood vessel using standard catheterization techniques through a remote vessel, eliminating the need for extensive and prolonged exposure of the diseased or injured vessels.

The endovascular approach carries several potential advantages. Firstly, it provides an easy and quick surgical approach to remote vessels. In the case of a vascular surgery it might provide access to both vessel sides (distal and proximal). Secondly, we hypothesize that the close proximity to blood vessel wall will result in a lower constriction-inducing current threshold and fewer adverse reactions, such as undesired muscle contraction as well as cardiac arrhythmias, which have been observed in pulsed treatment during irreversible electroporation^14^,^15^,^16^.

In this paper we introduce the concept of the novel approach of endovascular application of electrical pulses for vasoconstriction, and present a computer simulation study for the characterization of the applied electrical field and induced thermal heating. In order to investigate the effect of electrical pulses on vessels vasoconstriction, we performed computer based mathematical modeling studies using COMSOL Multiphysics for the characterization and the optimization of endovascular electrode configuration and pulse parameters. We modeled the porcine abdominal aorta using electrical and thermal properties reported in literature^17^,^18^,^19^ and investigated the resulting electric field induced by electrical pulses with parameters previously shown to induce constriction for several electrode configurations and varying inter-electrode distance. We then studied the treatment expected effect on the blood vessel and tissue by modeling and solving the bio-heat equation. Thermal damage can cause cell death and protein coagulation and induce irreversible vasoconstriction and undesired effects on the vessel^20^,^21^,^22^. Thus, estimating the temperature change due to the treatment under the different conditions is of great importance. Our results indicate that the most effective and safe configuration for endovascular induced vasoconstriction, is the configuration where two active electrodes and two return electrodes are located endovascularly and are spaced 13 mm each apart. In this configuration, we achieved the maximal and most homogenous electrical field on the vessel’s wall.

More importantly, our investigations reveal that endovascular electrical pulses can achieve high electric field with no increase in tissue temperature.

These results form the theoretical basis towards the development of endovascular stimulation as an efficient and safe vasoconstriction approach.

## Results

### Electric Field

Using COMSOL Multyphysics 4.4 software (COMSOL Inc., Sweden), we modeled the porcine abdominal aorta as a two dimensional rectangle domain with length of 250 mm, diameter of 12 mm and thickness of 1.5 mm^17^,^18^,^19^ surrounded by muscle tissue modeled as a rectangle domain of 200 mm length and 700 mm height. The vessel’s length was extended in order to observe collateral effects and avoid boundary conditions effect. To ensure that extending the model did not affect the results, a non-extended, 50 mm length artery model was also studied, and showed no significant differences in the electric field, as compared to the 250 mm arterial length model (supplementary Fig. S1).

**Figure 1:**
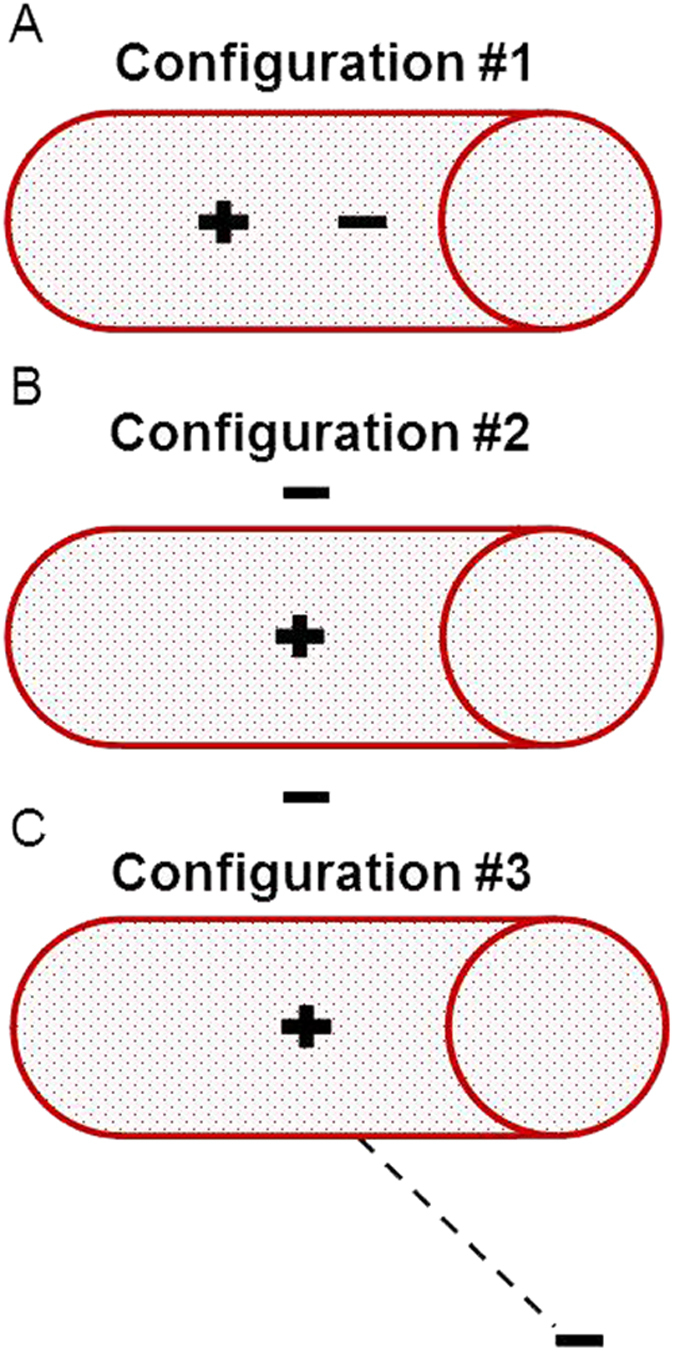
Geometric model and electrode configuration. The red cylinder is schematic illustration the abdominal aorta, with a diameter of 12 mm, length of 250 mm and thickness of 1.5 mm. (**A**) Endovascular configuration of electrodes of both active (+) and ground (−) electrodes, sized 1*1 mm. (**B**) endovascular active electrode with extravascular round ground. (**C**) Remote ground configuration with endovascular active and a large (15*150 mm) ground, situated 40 cm from the vessel.

Three basic electrode configurations were studied (Fig. 1).

**Figure 2:**
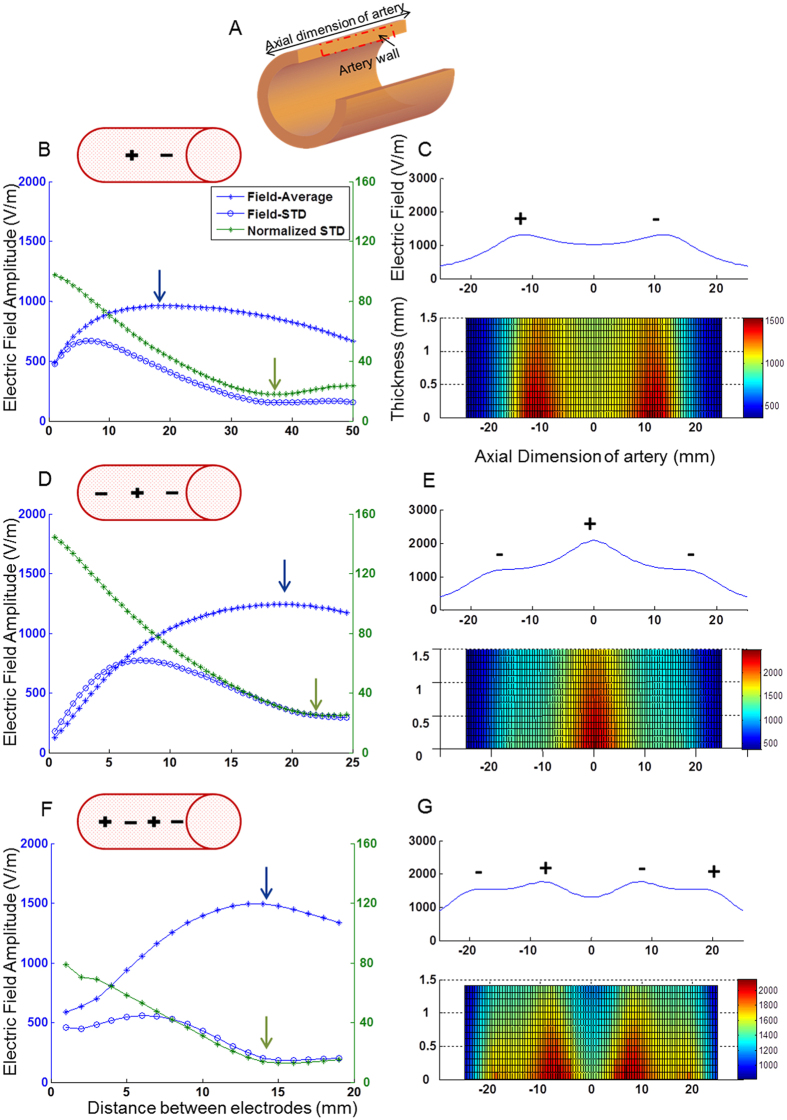
The electric field behavior, configuration #1. (**A**) Illustration of the cross section of the artery and the area over which the analysis was performed. (**B**,**D**,**F**) The average electric field on the artery sidewall, for various inter-electrode distances (blue asterisk), and its homogeneity (normalized standard deviation) (green), for each number of electrodes (2, 3 and 4). The blue and green arrows represent the area of maximal electric field and minimal standard deviation respectively. (**C**,**E**,**G**)- Upper trace- The average electric field profile on the artery sidewall at the optimal distance, where the electric field is at its maximum and the standard deviation is low. Lower trace- the electric field profile.

Configuration #1, was comprised of endovascular active and return electrodes, sized 1×1 *mm*^2^ (Fig. 1A). Configuration #2 was comprised of endovascular active electrodes sized 1 × 1 *mm*^2^ and two extravascular return circular electrodes with diameter of 3 mm, situated 1.5 mm away from the vessel wall (Fig. 1B). Finally, configuration #3 was comprised of an active endovascular electrode sized1 × 1 *mm*^2^ with a remote return, 40 cm away from the blood vessel and sized 15 × 150 *mm*^2^ (Fig. 1C). For each of the described configurations, simulations were initially performed with one active electrode, and for proceeding simulations the number of active electrodes was increased, with the electrodes equally spaced from each other. The electric current COMSOL physics model was solved for an electric pulse of 40 V with pulse duration of 10 ms and a repetition rate of 1 Hz, for each of the configurations. The model calculated the amplitude of the electric field along the blood vessel wall and adjacent tissue. We estimated the homogeneity of the electric field using the electric field standard deviation as a measure. In addition, we defined a safety measure by calculating the localization of the electric field on the area of interest, as defined by the ratio between the electric field on the artery wall and the tissue. The artery wall was defined as an area of 1.5 mm thickness and a length of 50 mm, as depicted in Fig. 2A.

**Configuration #1** was comprised of 2 endovascular electrodes, one being the potential, and the other return. Figure 2B shows the electric field amplitude induced by configuration #1 as a function of the inter-electrode distance. In this configuration, maximal average electric field (962 V/m) is obtained at an inter-electrode distance of 19 mm (Fig. 2B, blue arrow). However, at this distance, the electric field is not homogenous and is concentrated around the 2 electrodes as can be observed in Fig. 2C, as further revealed by high field STD. Maximal homogeneity of the electric field in this configuration, is obtained at an inter electrode distance of 39 mm (green arrow), more than two times larger than the distance where the maximal electric field is achieved. A significantly higher average electric field (1242 V/m) is achieved with three electrodes (an additional return electrode) at an inter-electrode distance of 19 mm (Fig. 2D, blue arrow). However, similar to the 2 electrodes version, there is a mismatch between maximal homogeneity (green arrow) and maximal electric field, which are reached at different electrode distances. However, when adding another active electrode (total of 4 electrodes- Fig. 2F), both maximal electric field on the artery wall (1495 V/m, blue arrow) and homogeneity (STDEV=14%, green arrow) are obtained with the same electrode distance of 13 mm (Fig. 2F).

Thus, in configuration #1, the 4 electrode version yields the optimal electric field in terms of maximal obtained field and homogeneity along the artery. The electric field profiles on the artery wall, for the optimal electrodes distances are shown Fig. 2(C,E,G) in both one dimension (upper traces) and two dimensions (lower traces). These figures further highlight the homogenous distribution of the obtained electric field in the 4 electrodes version (Fig. 2G) as compared to two or three electrodes. Figure 2G further illustrates that the electric field distribution is relatively concentrated on the inner side of the arterial wall and decreases toward the outer side of the artery, therefore contributing to the localization of the treatment, as will be discussed in the treatment localization section. The 4 electrode version was further investigated in the case of a constricted artery, showing that the electric field on the artery sidewall is further enhanced in this case by 25 percent, as compared to the non-constricted state (see supplementary Fig. S2).

**Figure 3:**
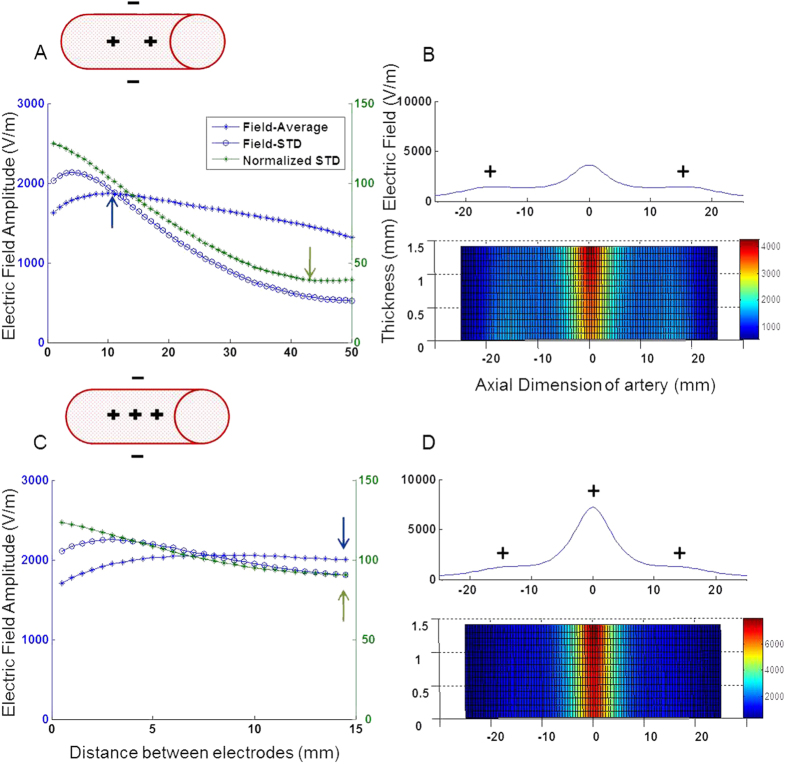
The electric field behavior, configuration #2. (**A**,**C**) The average electric field on the artery sidewall, for various inter-electrode distances (blue asterisk), and its homogeneity (normalized standard deviation) (green), for each number of electrodes (2 and 3). The blue and green arrows represent the area of maximal electric field and minimal standard deviation respectively. (**B**,**D**) Upper trace- The average electric field profile on the artery sidewall at the optimal distance, where the electric field is at its maximum and the standard deviation is low. Lower trace- the electric field profile.

**Configuration #2**. Figure 3 illustrates the electric field amplitude obtained by endovascular active electrodes with extravascular return electrodes at various inter-electrode distances (of the active electrodes). In the one active electrode with two extravascular return electrodes version, the electric field homogeneity was very low with STD as high as 126% (Table 1). In the two endovascular active electrodes version, the maximal average field is obtained at a distance of 11 mm between the electrodes and is 1885 V/m (Fig. 3A, blue arrow), where the homogeneity is still low (STDEV = 100%). However, the most homogenous field (minimal normalized standard deviation of 39%) is obtained at a different inter-electrode distance (45 mm apart- Fig. 3A, green arrow). In the three active electrodes version (Fig. 3C), although the maximal average field and the maximal homogeneity are obtained at the same inter-electrode distance, the homogeneity is poor (STD as high as 90%, Fig. 3C). To further highlight the lack of field homogeneity at the inter electrode distance for which the maximal field is obtained, the electric field profiles on the artery wall are shown Fig. 3B,D in both one dimension (upper traces) and two dimension (lower traces).

**Figure 4:**
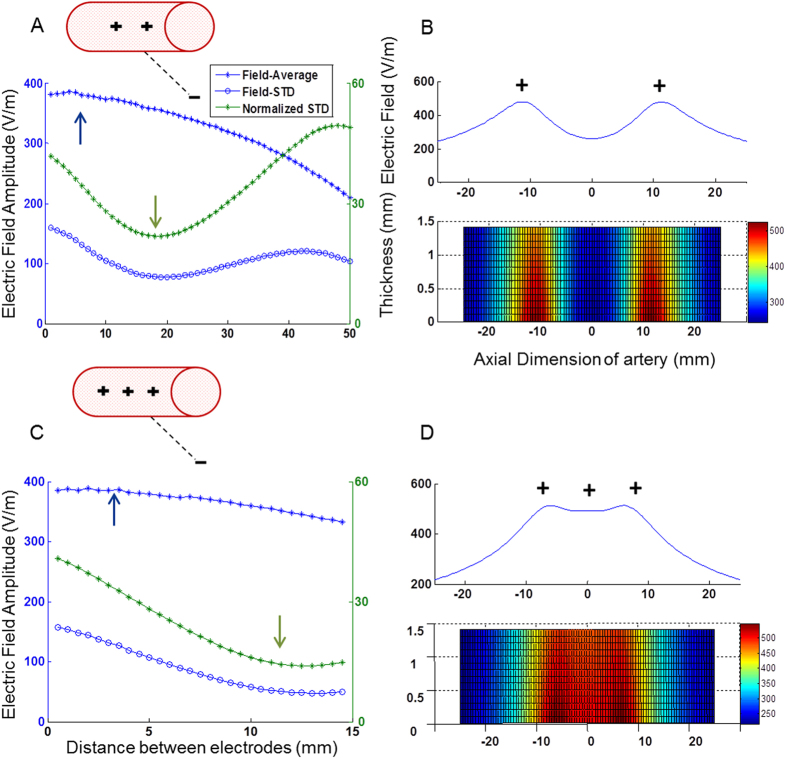
The electric field behavior, configuration #3. (**A**,**C**) The average electric field on the artery sidewall for various inter-electrode distances (blue asterisk), and its homogeneity (normalized standard deviation) (green), for each number of electrodes (2 and3). The blue and green arrows represent the area of maximal electric field and minimal standard deviation respectively. (**B**,**D**) Upper trace- The average electric field profile on the artery sidewall at the optimal distance, where the electric field is at its maximum and the standard deviation is low. Lower trace- the electric field profile.

**Configuration #3.** As can be observed in Fig. 4A,B upper trace this configuration was comprised of endovascular active stimulation with a remote, large return electrode. In the one active endovascular electrode version, the average electric field was as low as 371 V/m, much lower than the two previous configurations. Furthermore, the, electric field homogeneity was low, with a STD of 43% (Table 1). In the two active electrodes version (Fig. 4A), the maximal average electric field reached only 385.5 V/m (inter-electrode distance of 4 mm, blue arrow). Maximal electric field homogeneity was reached at a different inter-electrode distance (20 mm, normalized electric field STD=22%). In the 3 active electrodes version (Fig. 4C), the electric field is only slightly affected by the inter-electrode distance, with stable average field ranging between 300–400 V/m. A maximal electric field of 388.6 V/m was found at an electrode distance of 2 mm (blue arrow). Similar to the 2 electrodes version, maximal field homogeneity (Fig. 4C, green arrow) was found at a different inter-electrode distance (12.5 mm, normalized field STD=14%).

These data reveal that configurations #2 and #3, as opposed to configuration #1, are not suitable for effective endovascular clamping if maximal field value and field homogeneity are considered as parameters.

### Localization of Electric Field

We defined a localization value for estimating the localization of the induced electrical field on the arterial wall, which is important for treatment safety and effectiveness. We defined a localization value as the ratio between the average electric field on the artery wall and the tissue electric field (equation (1)). On the artery wall, the electric field was integrated along the arterial sidewall on an area of 1.5 mm (the sidewall thickness) ×50 mm, which is the area of interest in the case of electrical stimulation. On the tissue, the value of the electric field was measured on the whole upper part of the tissue, from the edge of the artery, to the edge of the geometry, in a 50 mm wide section. The ratio between the two averages was then defined as the localization value (LV):


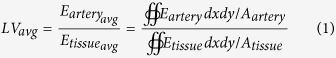


where *A* represents the surface area over which the electric field was averaged- 1.5 × 50 ***mm***^2^, and 100 × 50*mm*^2^ for the artery wall and for the tissue respectively.

For each electrode configuration, we evaluated the LV at the optimal inter-electrodes distance where the electric field is highest and most homogenous. In configuration #1, LV for 2, 3 and 4 electrodes version was found to be 5.56, 10.85 and 9.93, respectively (Table 1). In configuration #2, LV for 1, 2 and 3 active electrodes version was found to be 11.99, 7.18 and 8.78, respectively (Table 1).

In configuration #3, LV for 1, 2 and 3 active electrode was found to be 3.8, 3.27, and 3.29, respectively (Table 1).

**Figure 5:**
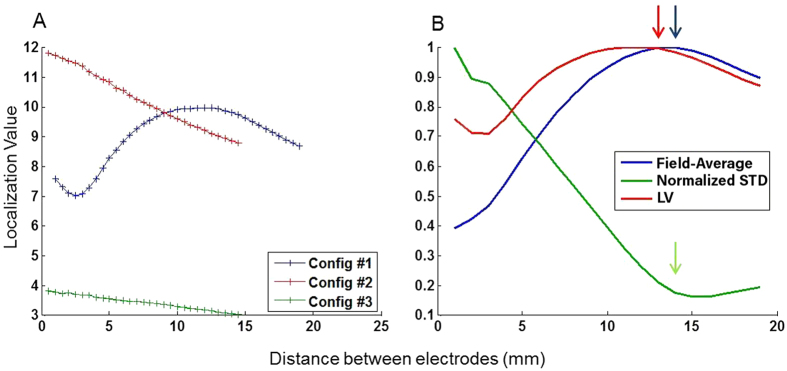
Localization Value (LV) and the optimal configuration. The ratio between the average electric field on the artery sidewall and on the tissue is presented for various inter-electrode distances. In order to choose the optimal configuration, the three parameters (electric field amplitude, homogeneousness and LV) were considered. (**A**) The LV for configuration #1 with four electrodes (blue), for configuration #2 with 3 electrodes and for configuration #3 with 3 electrodes is shown. (**B**) Normalized parameters of electric field, standard deviation and LV for the 4 electrodes version of configuration #1 are shown. The blue, green and red arrows point the maximal field average, minimal standard deviation and maximal LV respectively. Only in this version the optimal values of each parameter are at the same electrodes distance.

The LV of each configuration for the electrode number yielding the optimal field value and homogeneity as a function of inter-electrode distance is shown in Fig. 5A, further highlighting the superiority of configuration #1 over the two others. In this configuration, the optimal LV can be obtained at the same inter-electrode distance of 13 mm, as for the optimal electric field value, and the standard deviation (Fig. 5B, red, blue and green arrows respectively).

In order to choose the optimal configuration, we considered all three parameters: average electric field, homogeneity, and localization value as is summarized in Table 1. The obtained results suggest that the optimal electrode configuration is configuration #1, with 4 endovascular electrodes at an inter-electrode distance of 13 mm. In this configuration, the electric field was high, homogenous and was highly localized to the arterial wall (Fig. 5).

### Thermal Effect

**Figure 6:**
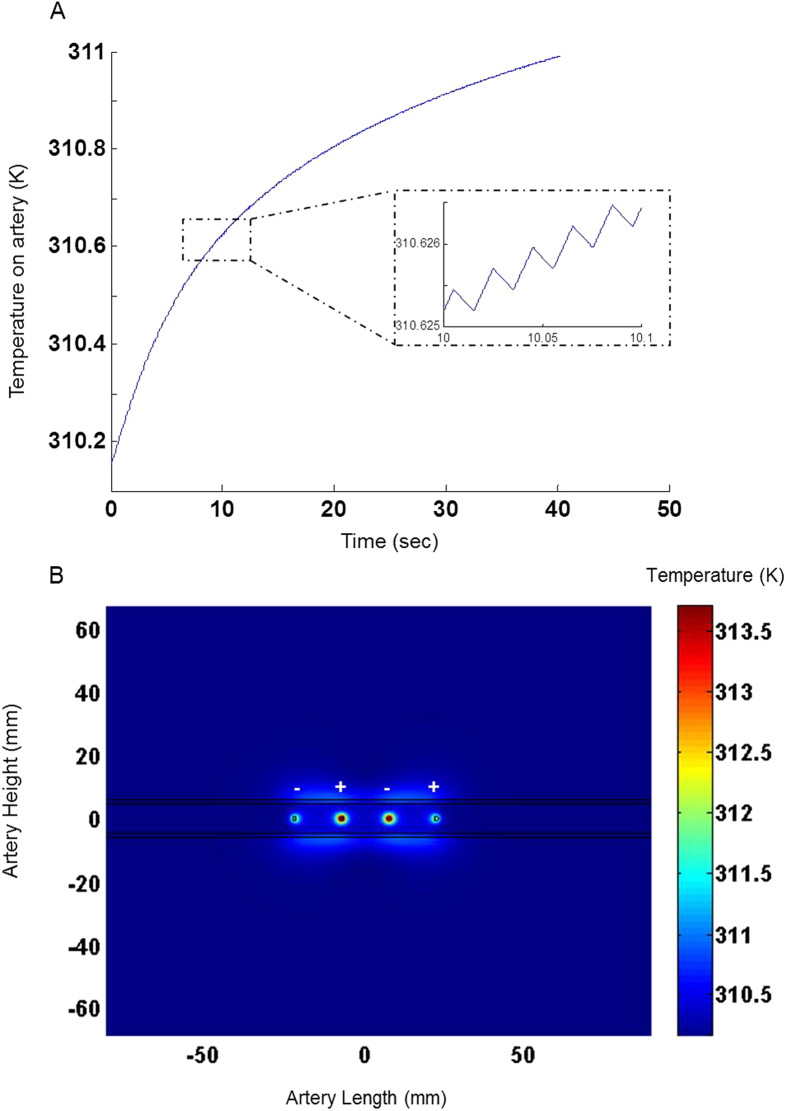
Temperature Behavior on the artery in a 40 V, 40 seconds stimulation session at 50 Hz repetition rate under 1% blood perfusion rate. (**A**) The temperature on the artery side wall during 40 seconds stimulation. Insert: zoom-in on the temperature behavior on the artery during the electrical pulses. (**B**) Temperature color-map on the whole geometry at the end of 40 seconds simulation.

The heating effect of the electric pulse was simulated for the optimal configuration for a pulse potential of 40 V, duration of 10 ms and frequencies of 1, 10 and 50 Hz, for a treatment duration of 40 seconds. These parameters were shown to cause 80% vasoconstriction in the rats’ femoral artery^12^, and were used here to investigate the effect on a larger blood vessel. Nevertheless, our model enables us to perform treatment planning for any given pulse amplitude, duration and repetition rate. In order to study the thermal effect on both normal and constricted blood vessel, simulation was repeated for three conditions of blood perfusion rates- 100% (58.34 s^−1^)^11^ 10% and 1% of the perfusion rate. The simulation shows that even at the worst case scenario, where the artery is stimulated at the highest pulse repetition rate (50 Hz) and is almost completely constricted (1% blood perfusion rate), the maximal temperature rise on the arterial wall was only 1.2 degrees. Figure 6A depicts the temporal temperature dynamics on the artery wall during the simulation, illustrating the small elevation in temperature. The insert shows the fluctuating temperature on the artery induced by the electric pulses. The temperature distribution on the artery at the conclusion of the treatment is depicted in Fig. 6B.

**Figure 7:**
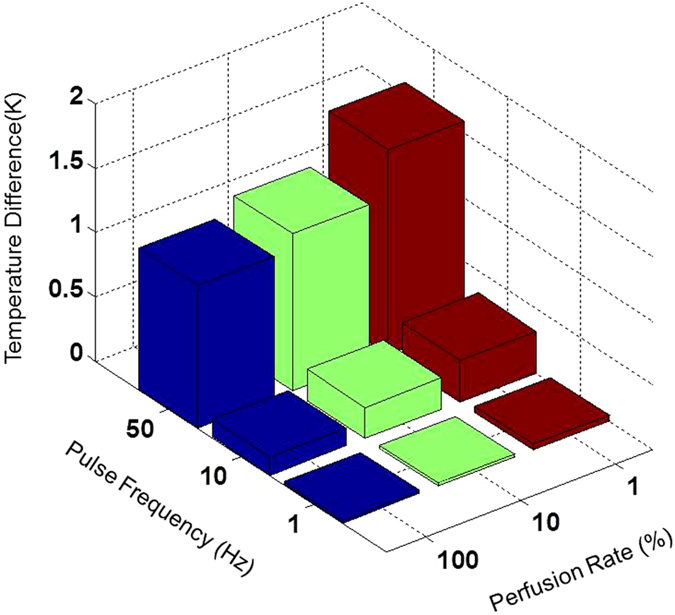
Maximal Temperature increase on the artery for a 40 seconds simulation session. Simulation of 40 V, 10 ms long pulses with a frequency of 1, 10 and 50 Hz. Three values of blood perfusion rate were analyzed- 100% (58.3 *s*^−1^), 10% and 1% perfusion rate, simulating a constricted blood vessel. The maximal temperature increase from the initial temperature on the artery sidewall, at the end of the session is presented.

Figure 7 summarizes the temperature increase for various pulse repetition rates under different flow rate conditions. It can be observed that even under high vasoconstriction conditions, where flow is very low and high repetition rate no significant increase in temperature is expected, highlighting the potential safety of endovascular electrically induced vasoconstriction.

## Discussion

In this simulation based study we present a novel approach for electrically induced vasoconstriction using endovascular electrodes. Short electrical pulses applied through extravascular electrodes have already been shown to induce vasoconstriction and to significantly decrease blood loss caused by blood vessel injury^11^,^13^. Moreover, the extent and duration of vasoconstriction was shown to be controlled by pulse amplitude, duration and repetition rate. In the present mathematical simulation study, we focused on the endovascular approach for stimulating blood vessel, showing that the use of endovascular electrodes can produce high, localized electric field and therefore have the potential to induce highly localized vasoconstriction effect. In our study we investigated the effect of the number of electrodes and their configuration on the induced electrical field while keeping the electric pulse parameters constant (using parameters which have already been proven to induce efficient vasoconstriction)^11^,^12^. We studied the electric field amplitude at the artery wall, the electric field homogeneity, localization and the resulting thermal heating. Our study reveals that, the optimal configuration was the 4 electrodes version of configuration #1, which yielded a localized, homogenous and high electrical field (Table 1 and Fig. 5B). Furthermore, when varying the number of endovascular electrodes and their configuration we observed that optimal parameters were obtained when the pulse was applied by four electrodes (2 active and 2 return) 13 mm apart. Significantly lower electric field amplitude and homogeneity were obtained in configurations with extravascular return electrodes (configuration #2, #3). As seen in Table 1, although the electric field in configuration #2, with 3 electrodes, is higher for a specific pulse potential, its homogeneity is very low (STD = 90%). It should be noted that the electric field amplitude can be easily increased by increasing the pulse potential, since the electric field intensity is linear to the pulse potential (which was arbitrarily modeled here as 40 V).

More importantly, we observed that configuration #3, which simulates the configuration currently used in many electro-surgical procedures^23^,^24^,^25^ yields a very low localization value compared with the other configurations. This should be an important consideration when deciding upon the desired electrodes configuration, especially when trying to avoid undesired muscle twitching and contraction^14^,^15^,^16^. Furthermore, the electrical field in configuration #1 is expected to be radially symmetrical, as opposed to the other configurations (where the linear external electrodes induce electric field asymmetry), thus offering the advantage of symmetric constriction. It should be noted that it is also possible to obtain a radially symmetrical electric field in configurations #2 and #3. This, however, requires the insertion of an encircling electrode, necessitating a challenging surgical dissection. More importantly, the increased arterial-wall electrical field observed in a constricted artery, (supplementary Fig. S2) suggests a positive feedback mechanism, which further enhances the effect of endovascular electrodes.

It should be noted that this model is a simplified model of a realistic artery comprised of different layers (intima, media and adventitia). Nevertheless, a more realistic artery model simulation yielded a similar electric field profile, as compared to the simple artery model (Supplementary Fig. S3). Similar results were also reported by Ivorra^26^.We believe that the effect of the endothelial layer in large vessels is small because of the relatively low electric resistance of gap-junction endothelia^27^,^28^,^29^.

By solving the bio-heat equation, we showed that despite the high and localized electric field obtained with the chosen configuration; minimal temperature rise is induced, thus avoiding potential thermal damage (Fig. 6). Tissue damage occurs when the tissue is exposed to temperatures higher than 42 °C, for a prolonged period of time^10^,^20^,^30^. In our study, we demonstrated that the maximal temperature rise in the artery sidewall was 1.2 at the worst case scenario (pulse repetition rate of 50 Hz, 1% blood perfusion rate).

Our model thus enables the conduction of a thorough treatment planning, by adjusting the configuration, location or number of electrodes for the desired electric field, corresponding to the vessel’s geometry.

In conclusion, we present here for the first time a novel approach for inducing blood vessel constriction by endovascular electrical stimulation. Our simulation shows that endovascular stimulation can offer a highly localized electric field with minimal thermal damage. This method may prove to be beneficial in both emergency and elective surgical procedures, where vascular constriction is desired in vessels with limited access.

## Method and Model

The goal of this study is to determine which electrode configuration will produce the maximal electric field with high homogeneity on the blood vessel’s walls, where the treatment targets, namely arterial smooth muscles and sympathetic nerve terminals^17^,^31^,^32^ are located, without producing thermal damage. The electric field and the temperature distribution induced by the pulses were modeled and solved for using finite element analysis using COMSOL Multyphysics 4.4 software (COMSOL Inc., Sweden).

### Geometric model

We simulated a large blood vessel, such as the porcine abdominal aorta^17^,^18^,^19^. Three basic configurations were modeled: configuration #1, with active and return endovascular electrodes (Fig. 1A); Configuration #2, with an active endovascular electrode, and two extravascular ground electrodes (Fig. 1B); and configuration #3, with an active endovascular electrode and a “remote ground”, placed on the outer boundary of the model, simulating a large skin electrode, routinely used in electro surgical procedures^23^,^25^,^33^,^34^ (Fig. 1C). Versions of the three configurations included one, two and three active electrodes, with an inter-distance varying from 1–20 mm.

### Electrical model

The electrical potential and electric field for each spatial point in the model were calculated using COMSOL’s electrical current physics model by solving the electric field equation:


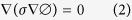


where *σ* is the electrical conductivity and ∅ is the potential at a specific location. The electric field was then calculated from the electric potential by taking the derivative:


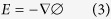


Electrodes were represented as separate domains of an electric potential boundary condition in which one (or more, according to the configuration) electrode had a positive potential, Vp, referred to as the pulse potential and the other was zero (the ground). The interface between the electrode and the blood was neglected, as previously done^11^,^13^. The outer boundaries of the muscle were set to an insulating condition in which


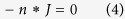


where n represents the unit outward normal vector and J represents the current density, and a continuity condition was applied to all other boundaries.

### Bioheat Model

After solving the field equation, the Joule heating (*p*) rate per unit volume (W/m^3^) caused by the electric field was calculated by


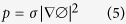


The Joule heat generated during the treatment was then added to the Pennes bioheat equation^35^


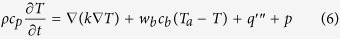


where *k* is the thermal conductivity of the tissue, *T* is the temperature, *w*_*b*_ is the blood perfusion, *c*_*b*_ is the heat capacity of the blood, *T*_*a*_ is the arterial temperature, *q*′″ is the metabolic heat generation, *p* is the electric heat generation, ρ is the tissue density, and *c*_*p*_ is the heat capacity of the tissue. A similar approach was modeled by Mandel *et al*.^13^, with the exception that in the current model the artery is exposed to the internal body temperature as opposed to the ambient temperature,. This was modeled by assigning a boundary condition of zero total heat flux assigned to the outer boundaries of the muscle, simulating a steady state situation. The initial temperature of the whole geometry was set to 37 ° (310.15 K). The problem was solved for an electric pulse of 40 V with pulse duration of 10 ms and a repetition rate of 1, 10 and 50 Hz, for 40 seconds sessions.

### Physical constants

The electrical properties of the blood vessel and the muscle were taken from an online database^36^ based on the work of 37 Gabriel *et al*. and are summarized in Table 2. The thermal properties of the tissue were taken from the literature^13^,^30^ and from the online data base of the Foundation for Research on Information Technologies in Society (IT’IS)^38^ and are summarized in Table 2. The metabolism was assumed to be 33,800 W m^−3^^39^. To account for the blood flow in non-constricted vessel, the perfusion rate inside the blood vessel was set to 58.34 *s*^−1^, corresponding to a flow rate of 1.575 L/min^11^,^40^ through a 6 mm diameter blood vessel (inner diameter), where the heated length is 50 mm. This length is the total length affected by the four electrodes, separated 15 mm from each other. We then simulated the same configuration with 10% and 1% of the perfusion rate, in order to investigate the effect on a constricted blood vessel. The electrodes in the model are of 304 stainless steel, similar to those used in Al-Sakere *et al*.^30^ and their electrical and thermal properties are shown in Table 2.

### Solution algorithm

The solution of the problem was performed by COMSOL Multiphysics 4.4 with MATLAB R2013b. The mesh consisted of 5000–8400 triangular elements (10400–16800 degrees of freedom), depending on the number of electrodes and on the geometry. The mesh size was optimized with respect to accuracy and solution time. The solution of the electrical problem was calculated for pulse duration of 10 ms, with a potential of 40 V and for the different electrode configurations. These pulse parameters were shown to cause 80% vasoconstriction in the rats’ femoral artery^12^ thus were used here to further explore their effect on a larger blood vessel. It should be expected that higher electric field will be needed to stimulate larger blood vessels. However, since the model is linear, the electric field amplitude can be increased by increasing the pulse amplitude. The solution of the electrical problem was obtained by using a direct linear solver (MUMPS). In every configuration, the electric field in each point on the geometry was calculated, followed by averaging of the electric field on the blood vessel’s upper wall (1.5 mm thickness) in a 50 mm section and on the tissue (50 mm width and 100 mm height from the outer boundary of the vessel). The average electric field along the artery wall thickness was calculated and presented along the arterial length. The standard deviation of the electric field on these areas was calculated as well, in order to depict the homogeneity of the field distribution along the artery. An “efficient” configuration was defined as the one with a high average electric field on the artery, and a low standard deviation value. Finally, as an additional parameter of treatment effectiveness and safety, a localization value (LV) was defined as the ratio between the average electric field on the artery wall to the average electric field on the tissue. The higher the LV is the more localized the treatment is to the blood vessel wall.

## Additional Information

**How to cite this article**: Kezurer, N. *et al*. Endovascular Electrodes for Electrical Stimulation of Blood Vessels for Vasoconstriction – a Finite Element Simulation Study. *Sci. Rep.* **6**, 31507; doi: 10.1038/srep31507 (2016).

## Supplementary Material

Supplementary Information

## Figures and Tables

**Table 1:** Summary of the electric field results.

Configuration	Optimal Inter-Electrode (mm)	Average Electric Field at optimum(V/m)	STD at optimum(%)	Localization Value
#1- 2 electrodes	25	950	32	5.56
#1- 3 electrodes	17	1233	37	10.85
#1- 4 electrodes (*)	13	1494	16.5	9.93
#2- 1 electrode	—	1400	126	11.99
#2- 2 electrodes	35	1579	46	7.177
#2- 3 electrodes	14.5	2001	90	8.776
#3- 1 electrode	—	371	43	3.8
#3- 2 electrodes	20	352	22	3.273
#3- 3 electrodes	10	360	16	3.286

The optimal configuration, combining the three parameters is marked with (*).

**Table 2:** Dielectric and thermal properties of the abdominal aorta and the electrodes used in the model.

	Blood	Aorta wall	Surrounding muscle	Electrode- Stainless steel
Electric conductivity σ (s/m)	0.7 (37)	0.25 (37)	0.25 (37)	2.22 × 10^6 (30)
Relative permittivity εr	1.4*10^3 (37)	1*10^7 (37)	1.4*10^7 (37)	1 (30)
Density ρ (kg/m^3)	1000 (13)	1102 (38)	1090 (38)	7900 (30)
Thermal Conductivity K (W/m*K)	0.52 (38)	0.46 (38)	0.49 (38)	14 (30)
Heat Capacity Cp (J/kg*K)	3640 (13)	3306 (38)	3421 (38)	477 (30)
Temperature (K)	310.15	310.15	310.15 (*)	310.15 (*)
Perfusion Rate (1/s)	58.34 (11)			

Marked with (*), is the temperature after reaching steady state, with a total heat flux of 0.
