# Supplementary material for: Endovascular Electrodes for Electrical Stimulation of Blood Vessels for Vasoconstriction – a Finite Element Simulation Study
Source: Sci Rep. 2016 Aug 18;6:31507. doi: 10.1038/srep31507 (PMC4989140; doi:10.1038/srep31507)
Supplement: Supplementary Information [file srep31507-s1.pdf]

Supplementary Information

Endovascular Electrodes for Electrical Stimulation of Blood Vessels for Vasoconstriction – a Finite Element Simulation Study

Noa Kezurer[1], Nairouz Farah[1], Yossi Mandel[1]

[1]Mina and Everard Goodman Faculty of Life Sciences, Optometry and Visual Science Track and Bar-Ilan's Institute for Nanotechnology and Advanced Materials (BINA), Bar-Ilan University, Ramat-Gan, Israel.

Corresponding Author:

Yossi Mandel, M.D., PhD, MHA

The Mina & Everard Goodman Faculty of Life Sciences

Bar Ilan University, Israel

Tel/Fax: +972-3-7384234

Email: yossi.mandel@gmail.com

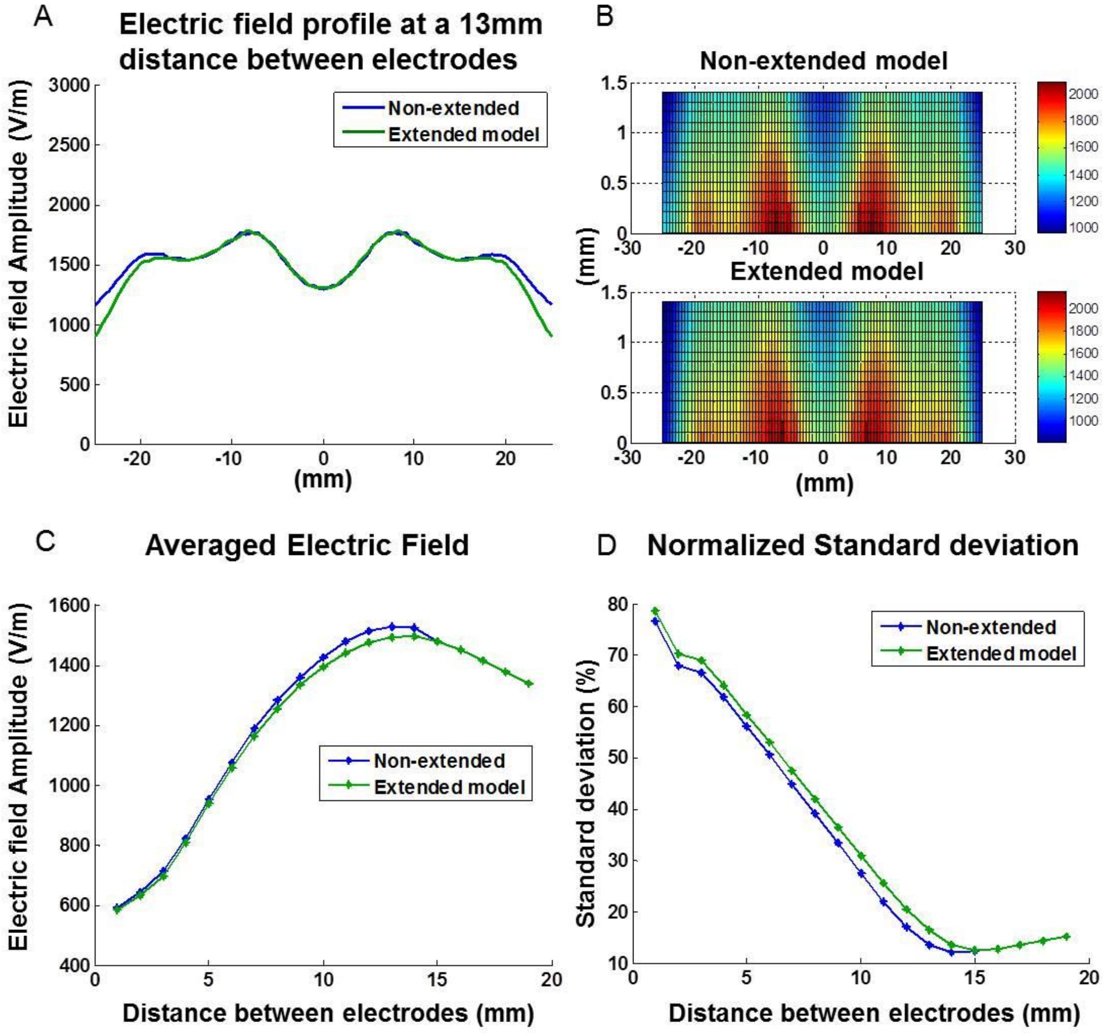

Supplementary Figure S1. Electric Field distribution -comparison between an extended and non-extended model for electrode configuration #1. The extended artery model (250 mm) and the non-extended (50mm) for electrode configuration #1 (which was found to be the optimal configuration) were compared. A- The average electric field profile on the artery sidewall at the optimal inter-electrode distance (13mm) for the extended (green) and non-extended (blue) model are very similar. B- The electric field profile on the artery sidewall at the optimal inter-electrode distance (13mm) for the extended (lower trace) shows high similarity to the non-extended (upper trace) model. C and D- The average electric field and the normalized standard deviation (respectively) on the artery sidewall shows high similarity for various inter-electrode distances for the extended (green) and non-extended (blue) model.

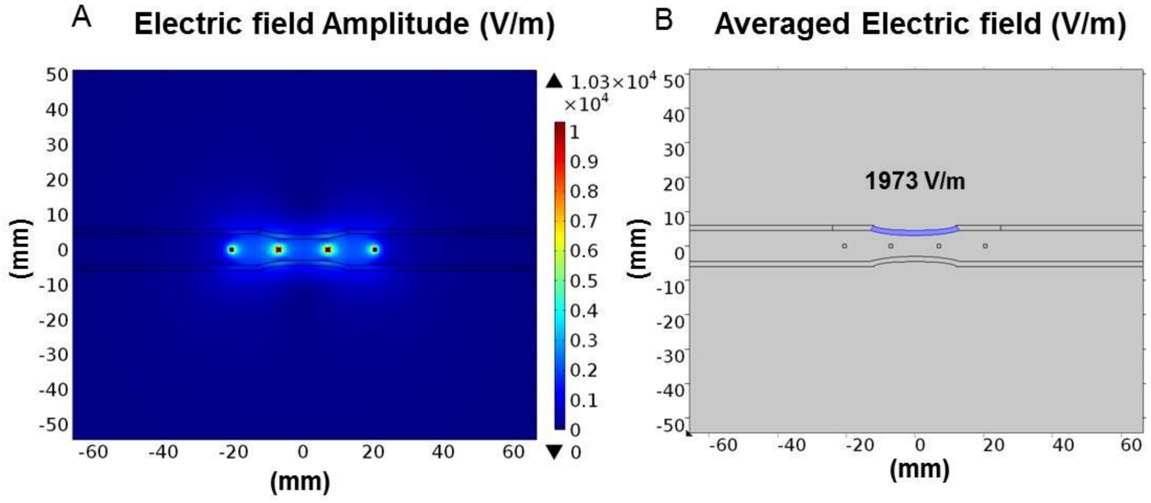

Supplementary Figure S2. The effect of constriction on electric field distribution. The artery was constricted by 33% in order to investigate the effect on the electric field in the 4 electrode version of configuration #1. A- The electric field distribution on the constricted artery is shown. B- The electric field was averaged on the upper sidewall of the artery at the constricted area. The average electric field on the constricted artery and was 1973 V/m, a 25% increase compared to the non-constricted artery.

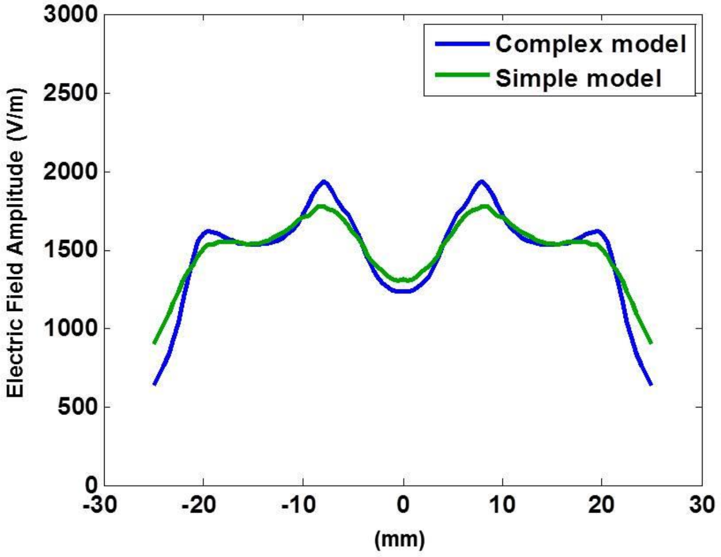

Supplementary Figure S3.Comparison of the average electric field profile in a complex and a simple artery model for the optimal configuration. The artery wall was modeled as a three-layered vessel (complex model) - intima, media and adventitia. The electrical properties of each layer were taken from the analytical work of Ivorra[1], and the average electric field profile was calculated for each model. The average electric field on the artery wall medial layer for the complex and simple model were 1474 V/m and 1494 V/m respectively (1.3% difference). The normalized standard deviation of the electric field for the complex and simple model were 20% and 16.5% respectively.

References

1. Ivorra, A. Incorporation of the Blood Vessel Wall into Electroporation Simulations Incorporation of the Blood Vessel Wall into Electroporation Simulations. (2016). doi:10.1007/978-981-287-817-5
